# Minimal weight loss related to a short fasting period causes superior mesenteric artery syndrome in a patient with amyotrophic lateral sclerosis: A case report

**DOI:** 10.1097/MD.0000000000020571

**Published:** 2020-07-02

**Authors:** Dong-Ha Kang, Sung Woon Baik, Yu Hui Won, Myoung-Hwan Ko

**Affiliations:** a Department of Physical Medicine and Rehabilitation, Jeonbuk National University Medical School; b Research Institute of Clinical Medicine of Jeonbuk National University-Biomedical Research Institute of Jeonbuk National University Hospital, Jeonju, Republic of Korea.

**Keywords:** amyotrophic lateral sclerosis, nutrition, superior mesenteric artery syndrome, weight loss

## Abstract

**Introduction:**

Superior mesenteric artery syndrome (SMAS) is rare cause of small bowel obstruction and is characterized by an extrinsic vascular compression of the duodenum. The most common cause of SMAS is known as rapid and significant weight loss.

**Patient concerns:**

A 61-year-old man who was diagnosed with amyotrophic lateral sclerosis and maintained a stable diet before admission. When the patient re-started feeding by gastrostomy tube after 5 days of therapeutic fasting due to gastric ulcer caused by gastrostomy tube irritation, he presented postprandial vomiting, abdominal distention, and tachycardia. Since fasting, his weight has been reduced by about 3 kg.

**Diagnosis:**

Based on clinical symptoms and radiological findings, diagnose of SMAS was finally made. Abdomen computed tomography confirmed decreased aortomesenteric distance and tubography confirmed gastric and proximal duodenum distension above the compressed part.

**Interventions:**

We performed jejunal tube insertion and the amount of feeding through the jejunal tube was gradually increased while maintaining parenteral nutrition.

**Outcomes:**

The presenting symptoms of the patient gradually improved. Follow-up abdomen computed tomography and tubography showed improvement in duodenal narrowing and stomach distension.

**Conclusion:**

SMAS should be considered when there is an abrupt observation of symptom of gastrointestinal obstruction in patients with predisposing condition such as a low body weight, even if the weight loss is relatively small.

## 1 Introduction

Typically, most patients diagnosed with amyotrophic lateral sclerosis (ALS) present with symptoms of dysphagia as the disease progresses. The pathophysiological mechanisms of dysphagia in ALS are known to be primarily associated with the progressive degeneration of the excitatory and inhibitory corticobulbar pyramidal fibers that control the bulbar swallowing center.^[1]^ Recently, gastrostomy has become more widely used for patients with ALS because of its safety for long-term enteral nutrition.

Superior mesenteric artery syndrome (SMAS) is an uncommon cause of small bowel obstruction and is characterized by an extrinsic vascular compression of the duodenum; particularly, compression between the abdominal aorta and overlying superior mesenteric artery underlies this disease. This compression is related to anatomical/mechanical factors and acute or chronic reduction of the retroperitoneal fat pad. To date, many studies have reported predisposing conditions that lead to the development of SMAS. These conditions are commonly associated with marked weight loss or a hypercatabolic state.^[2]^ Furthermore, congenital anomalies and patient conditions, such as postoperative conditions, may contribute to SMAS development.^[3]^

Herein, we introduce the case of a male patient with ALS who developed SMAS acutely despite a relatively small weight loss after a short 5-day fasting period.

## 2 Case report

A 61-year-old man who was diagnosed with bulbar ALS was admitted to our hospital for evaluation and management of blood backflow through a gastrostomy tube. He had been diagnosed with ALS 26 months before admission and had undergone gastrostomy 6 months before admission. Additionally, 1 month before admission, he started full tube feeding and maintained a stable diet of 500 mL per meal. At the time of admission, he was 176 cm tall and weighed 43 kg (body mass index [BMI]: 13.88 kg/m^2^); his weight was similar to that measured 6 months before admission. He was using the home ventilator via tracheostomy tube full-time for 24 hours and all his limb muscles were generally measured as trace grade on the manual muscle test. Esophagogastroduodenoscopy was performed, and an acute gastric ulcer with recent bleeding was identified. The gastrostomy tube was removed because irritation by the tube was thought to be the cause of bleeding. The patient underwent therapeutic fasting for 5 days, and a high-dose proton pump inhibitor was administered intravenously.

After 5 days of fasting, the patient resumed enteral feeding through a nasogastric tube. Considering the healing process of the gastric ulcer, 100 cc of meal was fed through the nasogastric tube; this was increased to 200 cc per meal after 2 days. Since the patient had consumed about 1500 cc of meal per day before admission, the remaining nutrients were supplied via parenteral nutrition. A follow-up endoscopic examination confirmed a healing state of the gastric ulcer, and we re-inserted the gastrostomy tube 7 days after resuming the meal through the nasogastric tube (or 12 days after previous gastrostomy tube removal). The volume of diet administered through the gastrostomy tube was also initiated at 100 cc per meal and gradually increased. However, when the feeding amount reached 200 cc per meal, the patient presented postprandial vomiting, abdominal distention, and tachycardia. Because of these symptoms, it was difficult to increase the amount of enteral feeding volume supplied to the patient. Therefore, we adjusted the amount of the feeding and maintained it at 100 cc per meal inevitably.

**Figure 1:**
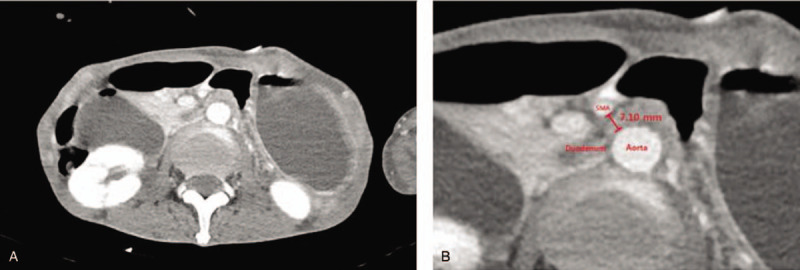
Abdominal CT findings at the time of diagnosis. (A) The second portion of the duodenum narrowed abruptly at the site between the aorta and the superior mesenteric artery. Near obstruction and severe dilatation of the stomach and proximal duodenum were observed. (B) The distance between the SMA and aorta was 6.23 mm, which is shorter than the normal range. CT = computed tomography, SMA = superior mesenteric artery.

**Figure 2:**
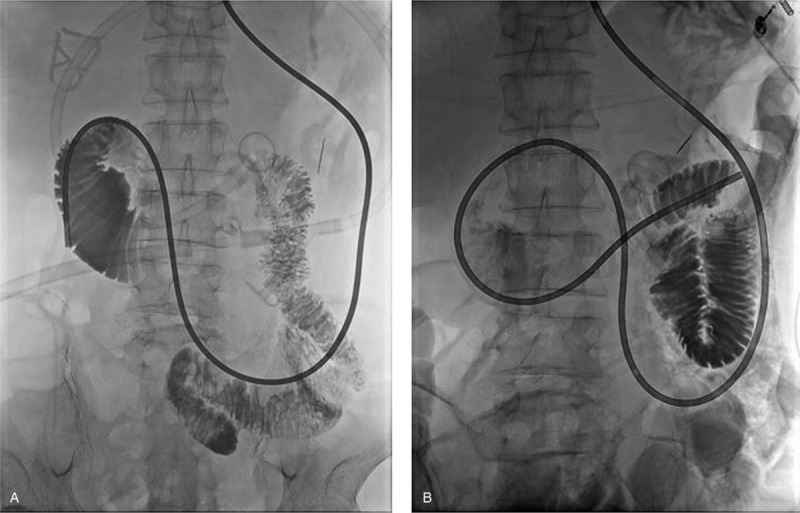
Tubography findings before and after successful therapeutic jejunal tube insertion. (A) In comparison to the distal duodenum, the proximal duodenum was dilated. A therapeutic feeding tube could not be advanced to the jejunum due to severe gastric retention. (B) The therapeutic feeding tube was successfully positioned in the jejunum, beyond the obstruction, after the gastric retention was improved.

Since these symptoms were not temporary, we performed computed tomography (CT) of the abdomen. We found that the second portion of the duodenum had become narrower between the aorta and the superior mesenteric artery. The patient also had severe stomach and proximal duodenum distension (Fig. 1A). In addition, the distance between the superior mesenteric artery and aorta was 7.10 mm, which is shorter than the normal range (Fig. 1B). Based on clinical symptoms and radiological findings, we were able to diagnose SMAS. The body weight was re-measured 5 days after fasting and it had reduced from 43 kg (BMI: 13.88 kg/m^2^) to 39.95 kg (BMI: 12.89 kg/m^2^). We performed jejunal tube insertion along with tubography and observed dilatation of the duodenum and severe gastric retention. Gastric retention was so severe that we could not advance the tube to the jejunum and it was placed in the second portion of the duodenum (Fig. 2A). Gastric retention was improved; 6 days later, the jejunal tube was repositioned beyond the gastric obstruction (Fig. 2B). Then, the amount of feeding through the jejunal tube was gradually increased while maintaining parenteral nutrition. Over the next 6 weeks, the presenting symptoms of the patient gradually improved. Follow-up CT of the abdomen and tubography were performed to observe improvement in duodenal narrowing and stomach distension. The dilatation of the stomach and duodenum, which was observed in previous examinations, was no longer observed, and tubography showed a passage of contrast through the duodenum without contrast stagnation. After these follow-up examinations, the patient could maintain diet through the gastrostomy tube again, and he tolerated 500 cc of diet at discharge. His body weight at the time of discharge was 43 kg, which was the body weight at the time of admission.

The evaluation finding included in this case study did not contain identifiable patient's personal information, and this case study involves no more than minimal risk to human subjects. For these reasons, this study was approved a waiver of informed consent from the institutional review board of the Jeonbuk National University Hospital (CUH 2019-10-024).

## 3 Discussion

This report presents a rare case of acute SMAS that developed after a relatively short fasting period of 5 days in a patient with ALS. To our knowledge, a systematic review has been conducted on SMAS, but few studies have quantitatively determined the correlation between weight loss or fasting duration and the risk of SMAS. However, case reports or case series studies on patients diagnosed with SMAS reported that these patients with various diseases lost 28.6% to 55% of their body weight, which translated to 16 to 50 kg of weight loss.^[4–10]^ It has been previously reported that patients diagnosed with ALS developed SMAS symptoms^[11,12]^; however, these patients did not undergo a short-term fasting state and were not receiving sufficient nutrition through a gastrostomy tube. In our case, on the other hand, the patient had a stable enteral feeding regimen (500 cc per meal) for several months. Furthermore, the fasting period was short (5 days) and the measured weight loss during this period was smaller than that previously reported (3.05 kg, 7.1% of his weight).

Pathophysiology of SMAS is related to anatomical position of duodenum which is located between aorta and superior mesenteric artery. The duodenum crosses the aorta and superior mesenteric artery, producing an acute angle with the aorta. The superior mesenteric artery is sustained by the uncinated process of the pancreas and left renal vein and forms an acute angle with the aorta. These structures, including the superior mesenteric artery, are enveloped by mesenteric and retroperitoneal fat. Rapid weight loss may lead to losses of mesenteric and retroperitoneal fat. If the distance and angle between the superior mesenteric artery and aorta are decreased as a result, the duodenum, which passes between them, is compressed and SMAS may occur.^[3,13]^ As shown in a previous study, a low BMI and rapid weight loss are important risk factors for developing SMAS.^[3]^ Other predisposing factors for SMAS include prolonged supine positioning, immobilization, and decreased abdominal tone; all of these factors are common occurrences among patients with quadriplegia.^[2,14–16]^ Our patient was considered to have a relatively high risk of developing SMAS because of a very low BMI and immobility due to quadriplegia. The patient was stable at home but had a body weight of 43 kg and a very low BMI. Therefore, the muscle mass and percentage of body fat were most likely very low compared to those of patients with normal weight. Our case report suggests that patients with low BMI who have the risk factors described above may develop SMAS, even after a relatively short fasting period and small weight loss.

Patients with SMAS present with symptoms that are suggestive of gastrointestinal obstruction, including persistent and recurrent postprandial nausea, emesis, epigastric fullness and bloating, and abdominal pain.^[2,3]^ In addition to clinical symptoms, various radiological examinations, such as X-ray studies, CT, or ultrasonography, can be used to diagnose SMAS. These examinations can demonstrate the following findings:

(1) dilatation of the proximal duodenum with or without gastric dilatation,
(2) failure of a contrast passage beyond the obstructive part of the duodenum with a cut-off, and
(3) severe delay of the gastroduodenojejunal transit time.^[13]^

Vascular abnormalities are well-delineated by measuring the aortomesenteric angle and distance through CT; the normal ranges for these measurements are 25° to 60° and 10 mm to 28 mm, respectively.^[17–19]^ An aortomesenteric artery angle of ≤25° is considered to be the most sensitive measure of diagnosing SMAS, particularly if the aortomesenteric distance has gradually decreased to ≤8 mm.^[20,21]^

SMAS is first treated conservatively if the case does not involve an aneurysm or other pathological conditions that immediately require surgical exploration, and conservative treatment has been increasingly successful as a definitive treatment.^[2,22]^ As conservative treatment, electrolyte imbalance and fluid deficiency are corrected, and weight gain is promoted to increase the aortomesenteric angle and facilitate the recovery of retroperitoneal fat tissue. To this end, administration of enteral jejunal tube feeding and parenteral nutrition support, as in the case of our patient, may be effective.^[3,13]^

The diagnosis of the SMAS is challenging and often delayed because of its insidious and rare presentation. If symptoms of gastrointestinal obstruction are observed abruptly in high-risk patients, as in this case, SMAS should be considered. Although SMAS is not as common and is generally diagnosed after excluding other diseases that cause the same symptoms, it still should be considered. Early diagnosis is necessary to avoid severe complications of SMAS, such as dehydration, metabolic imbalance, respiratory distress syndrome, or gastric perforation.

Based on this case, when bedridden patients with low BMI and malnutrition state are inevitably required to fast (ie, gastric bleeding due to ulcer), SMAS needs to be considered. Furthermore, it is necessary to minimize the fasting period as much as possible. Daily weight measurements may be appropriate to avoid an unexpected and abrupt weight loss.

## Author contributions

**Conceptualization:** Dong-Ha Kang, Yu Hui Won.

**Data curation:** Dong-Ha Kang, Yu Hui Won.

**Formal analysis:** Dong-Ha Kang, Yu Hui Won.

**Funding acquisition:** Myoung-Hwan Ko.

**Investigation:** Dong-Ha Kang, Yu Hui Won.

**Methodology:** Dong-Ha Kang, Yu Hui Won.

**Supervision:** Myoung-Hwan Ko, Yu Hui Won.

**Visualization:** Dong-Ha Kang.

**Writing – original draft:** Dong-Ha Kang, Sung Woon Baik.

**Writing – review & editing:** Myoung-Hwan Ko, Yu Hui Won.
